# Practical Considerations for the Management of Cushing's Disease and COVID-19: A Case Report

**DOI:** 10.3389/fendo.2020.00554

**Published:** 2020-09-11

**Authors:** Federica Beretta, Francesca Dassie, Matteo Parolin, Federico Boscari, Mattia Barbot, Luca Busetto, Roberto Mioni, Eugenio De Carlo, Carla Scaroni, Francesco Fallo, Roberto Vettor, Pietro Maffei

**Affiliations:** ^1^Clinica Medica 3, Department of Medicine, University of Padua, Padua, Italy; ^2^Division of Metabolic Diseases, Department of Medicine, University of Padua, Padua, Italy; ^3^Endocrinology Unit, Department of Medicine, University of Padua, Padua, Italy

**Keywords:** SARS-Cov-2, Cushing's disease, pituitary, rare diseases, hypercortisolism

## Abstract

**Introduction:** Italy, since the end of February 2020, is experiencing the corona virus disease 2019 (COVID-19) pandemic that may present as an acute respiratory infection. We report on COVID-19 pneumonia in the context of a complex case of Cushing's disease (CD).

**Case Report:** A 67-year-old man with CD, who was admitted to our hospital, presented with signs and symptoms of adrenal insufficiency with persistent hypotension and glycemia toward the lower limits. We progressively withdrew almost all treatments for diabetes and CD (pasireotide and metyrapone), and i.v. hydrocortisone was necessary. A tendency to hyperkalemia was probably associated to enoxaparin. We summarized the many possible interactions between medications of Cushing's syndrome (CS) and COVID-19.

**Conclusion:** Adrenal insufficiency might be a clinical challenge that needs a prompt treatment also in CS patients during COVID-19 infection. We should consider the possibility to titrate or temporary halt medical therapies of CS in the context of COVID-19 infection. Unexpected hyperkalemia in CS patients under treatment with heparin might be the signal of aldosterone suppression.

## Introduction

The severe acute respiratory syndrome coronavirus 2 (SARS-CoV-2) is the cause of a pandemic disease, called corona virus disease 2019 (COVID-19), which is currently affecting the world's population (1). The clinical presentation spectrum of COVID-19 is heterogeneous, ranging from a flu-like syndrome to severe pneumonia, not infrequently leading to acute respiratory distress syndrome and requiring intensive care support (2). COVID-19 can result in systemic inflammation, multi-organ dysfunction including the cardiovascular system, and venous thromboembolic events (3). Currently, no specific medication is recommended to treat SARS-CoV-2. There are studies supporting the use of corticosteroids at low-to-moderate dose in critically ill patients with coronavirus infection (2). In addition, an early and prolonged pharmacological treatment with low molecular weight heparin is highly recommended. Other treatments include antiviral, antimalarial, and monoclonal antibodies that target the IL-6 pathways (4).

The SARS-CoV-2 enters the pneumocyte using the ACE2 as a receptor, which is expressed in various tissues including the adrenal and pituitary gland (5). Autopsy studies on patients who died during the SARS outbreak in 2003 showed a direct cytopathic effect of the virus in the adrenal glands (6). COVID-19 infection may severely affect people with diabetes, obesity, malnutrition, Cushing's syndrome (CS), and adrenal insufficiency. In particular, a recent statement and several expert consensuses have been commissioned by the European Society of Endocrinology (ESE) as a clinical guidance on the management of endocrine conditions in the context of the COVID-19 pandemic (7–9).

Central obesity, hypertension, glucose intolerance or diabetes, proximal muscle wasting and weakness, and susceptibility to infections are frequently observed in CS patients (10). The disorder might have a high mortality rate if inadequately controlled (11). Vascular disease is the main cause of death in CS patients, and the risk of cardiovascular and cerebrovascular events is greater as compared with that of the general population. In addition, infections are among the most common cause of death prior to the start of any treatment of CS and during the follow-up. Several treatment options are available for CS patients; however, some of them might be challenging especially in the context of COVID-19 infection (12).

As stated in the ESE guidance on Cushing's management during COVID-19, the document was not intended to determine an absolute standard of medical care, and professionals need to consider individual circumstances (8). Direct experience of CS patients and COVID-19 infection can test and enrich this clinical guidance. Here, we report on a patient with active CD that was hospitalized for COVID-19 infection associated to hypotension and abdominal pain. Once more, this case emphasizes the need for careful clinical vigilance and management of CS also at the time of COVID-19. New practical aspects regarding CS medications and interactions with COVID-19 therapy have been summarized.

## Case Report

A 67-year-old man with CD was hospitalized by the end of March 2020 until April 29, 2020 with a COVID-19 pneumonia, diagnosed by a nasopharyngeal swab (13), presenting in a week before admission, with symptoms of fatigue, hyporexia, hypotension, dry cough, low-grade fever, and abdominal pain.

The past medical history of this patient was already reported in detail elsewhere (14). In short, he was operated for a relapsing adrenocorticotropic hormone (ACTH)-silent non-functioning pituitary macro-adenoma in 1993 and 2003 (transphenoidal and transcranial, respectively). The latter surgical procedure was complicated by a *Haemophilus influenzae* meningitis. He was also submitted to radiotherapy, and he had been on replacement therapy for hypopituitarism for several years (l-thyroxine and cortone acetate). Since 2013, cortone acetate was suspended owing to a biochemical and clinical picture of ACTH-dependent hypercortisolism, and he started pasireotide (s.c. and then monthly i.m. injections) and cabergoline to control CD. A regrowth of the pituitary adenoma was observed, and in 2017, he was operated on for a right orbital compression by a mass that resulted to be ACTH positive at immunohistochemistry. On May 2018, metyrapone was added (stopped for a few months in 2019 and restarted on January 2020). Sitagliptin and metformin were introduced on June 2018 to control diabetes. He also underwent several chemotherapy cycles from 2013 to 2015 with temozolomide. In 2018, he was treated with pembrolizumab early then switched to fotemustine, which was continued until January 2020 when he received two new cycles of temozolomide for the progressive enlargement of tumor size (the last treatment was on January 31). On February 2020, there was evidence of a deep venous thrombosis on the right upper arm associated with a peripherally inserted central catheter, which was treated with enoxaparin 8,000 UI s.c. x2/daily.

The COVID-19 pulmonary infection was treated with low-flow oxygen therapy, azithromycin for 6 days, ceftriaxone for 12 days, and hydroxychloroquine for 11 days (15). Parenteral nutrition was assured by a jugular central venous catheter. We initially maintained previous treatments with levetiracetam, proton pump inhibitors, vitamin D, l-thyroxine (100 mcg daily), cabergoline (0.5 mg daily), metyrapone (500 mg daily at bedtime), and sitagliptin (50 mg daily). Metformin, aspirin, and pasireotide LAR 40 mg were suspended on admission.

**Figure 1:**
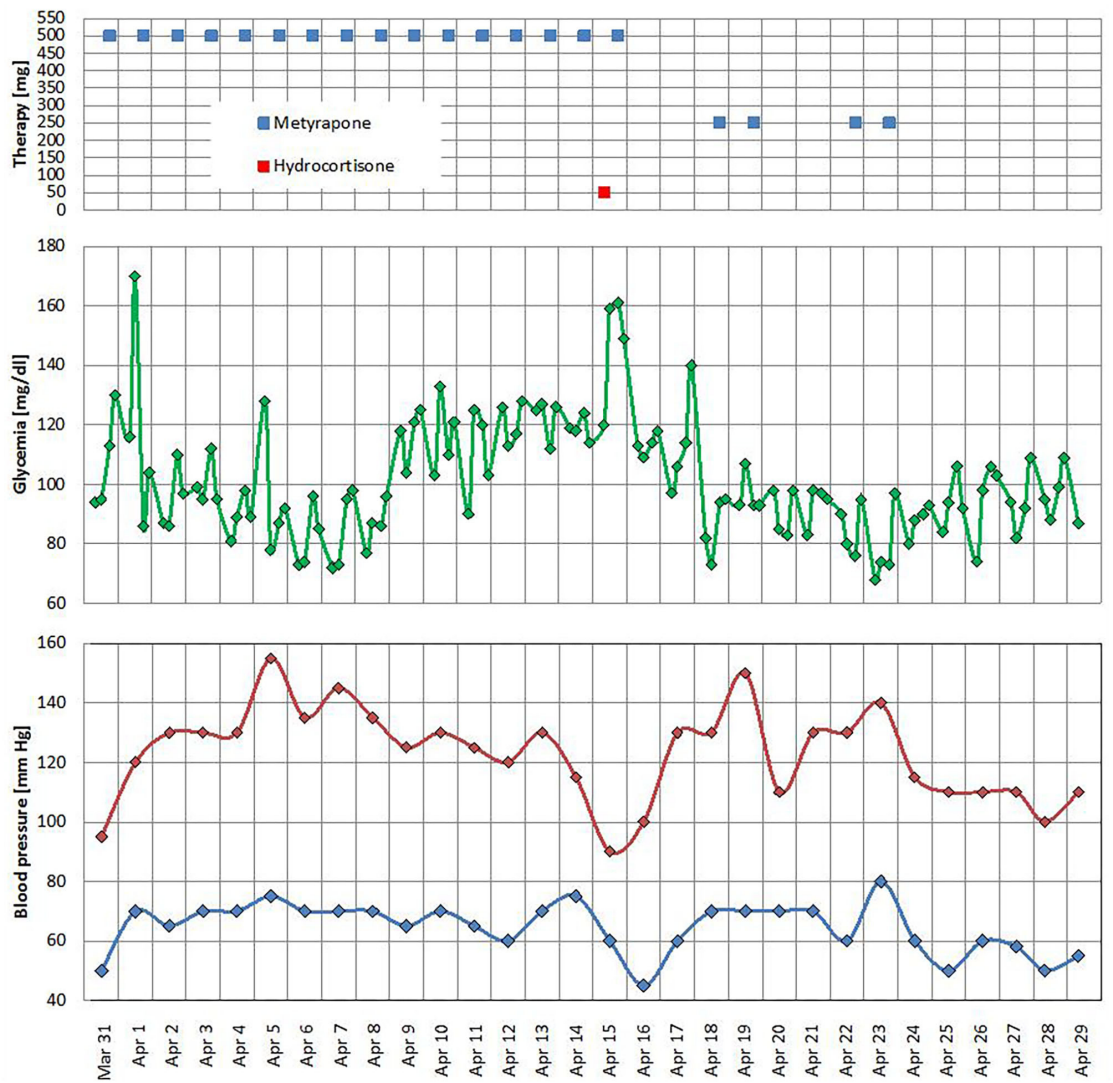
Metyrapone and hydrocortisone treatments, glucose level, and office blood pressure profile.

After 2 weeks of hospitalization, the patient was apyretic and eupnoic. The chest X-rays ameliorated, and oxygen support was stopped. All antidiabetic medications were suspended in the early days of hospitalization owing to the good glucose control; however, in the mid of hospitalization, we observed a persistence of symptomatic hypotension with hypoglycemia, and in suspicion of adrenal insufficiency (blood pressure 90/60 mmHg), we withdrew metyrapone and i.v. hydrocortisone 50 mg was promptly prescribed (Figure 1). Once blood pressure improved, a lower dose of metyrapone was restarted (250 mg/daily); however, after a few days, it was stopped again with maintenance of normal blood pressure and glucose levels (Figure 1).

At blood test on admission, there was an elevation of several inflammatory indexes [C-reactive protein (CRP) 57 mg/l], D-dimer (943 μg/l), and lactate dehydrogenase (284 U/l). He had a chronic leukopenia with lymphocytopenia probably owing to the recent chemotherapy. No alteration of coagulation markers in kidney and liver function was found. A tendency to hyperkalemia was detected during the whole hospitalization. Blood test results are shown in Table 1. The ACTH levels before admission ranged between 425 and 407 ng/l [normal values (n.v.) 10–50]; the day after we suspended metyrapone, ACTH was 424 ng/l, while at discharge, it ranged between 286 and 333 ng/l. The morning plasma cortisol (n.v. 138–690 nmol/l) at the time we suspended the metyrapone was 306 nmol/l and was in the range of 272–259 nmol/l at discharge without any specific treatment for CS, except cabergoline. The 24-h urinary-free cortisol (UFC; n.v. 16–168 nmol/24 h at mass spectrometry) before hospitalization was 315 nmol/24 h (December 2019) and 104 nmol/24 h (February 2020). At admission, the UFC was very low (3 nmol/24 h) and low normal by the end of hospitalization (34 and 14 nmol/24 h at discharge). A single plasma aldosterone level at discharge was 75.4 pmol/l (n.v. 48.7–643).

**Table 1:**
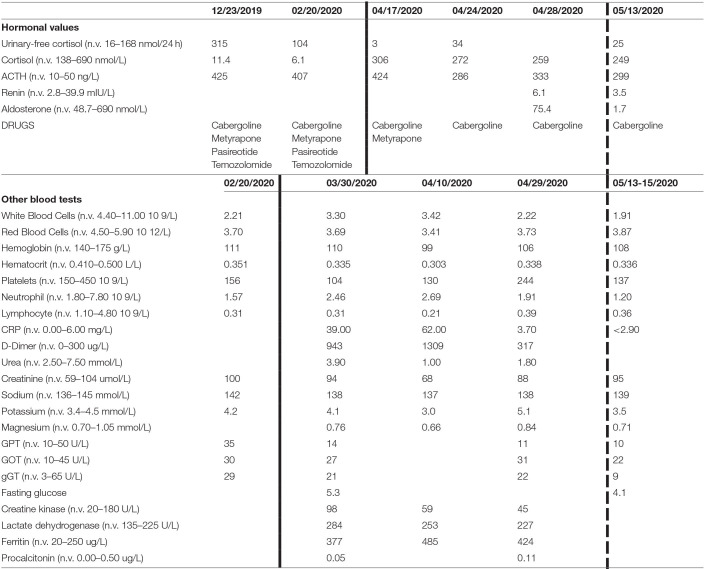
Blood tests results.

*The bold line indicates the beginning of hospitalization; the broken line indicates the end of hospitalization*.

**Figure 2:**
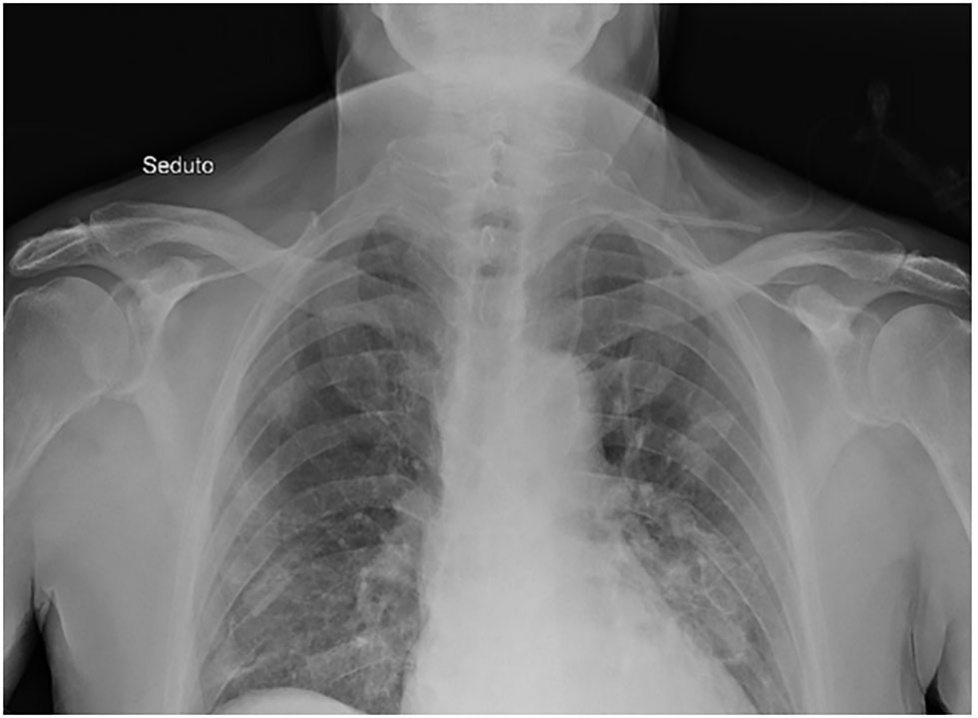
Chest X-ray. Chest X-ray description: small peripheral hazy opacity in medium field and more evident opacities at the medium-lower fields of both lungs, especially on the left side.

We did serial chest X-ray to follow the progression of lung infection. The chest X-ray at admission described small peripheral hazy opacity in medium field and more evident opacities at the medium-lower fields of both lungs, especially on the left side. Repeated chest X-rays evidenced the progression of bilateral pneumonia (Figure 2). The echocardiogram showed a left ventricular hypertrophy (FE 77%) and an estimated pulmonary pressure of 30 mmHg. Venous ultrasound of the upper right limb confirmed the persistence of thrombotic residues, so we continued the anticoagulant therapy with enoxaparin 8,000 IU s.c. twice daily for weeks and switched to edoxaban a few days before discharge.

The patient was eventually discharged in a rehabilitative facility suggesting a close endocrinological follow-up. Two weeks after discharge, SARS CoV-2 nasopharyngeal swab resulted negative. The pituitary MRI showed a slight reduction of tumor diameters. Cortisol was not suppressed after dexamethasone 1 mg while on treatment with cabergoline.

## Discussion

Notwithstanding the clinical challenge of CS, the prognosis was good, and eventually, we have been able to discharge this patient in a rehabilitation service. The pharmacological treatment of CS in association to the COVID-19 medications should take into account many possible interactions that we briefly summarized in Table 2.

**Table 2:** Cushing's syndrome drugs' characteristics and possible interaction with COVID-19 medications.

**Drug**	**Mechanism formulation dose**	**Side effects**	**Possible interactions with covid-19 drugs**
**Drugs with central pituitary action**			
Pasireotide^*^	Multi-ligand somatostatin receptors agonist 0.3, 0.6, and 0.9 mg, subcutaneous Recommended dose: 0.6 mg bid Maximum dose: 0.9 mg bid	Hyperglycemia Gastrointestinal side effects Hypocortisolism QTc prolongation Hypokalemia Liver dysfunction	Hydroxychloquine and Chloroquine may be used with attention on hypokalemia. Hydroxychloquine, Chloroquine, Azithromycin, Lopinavir, and Ritonavir prolong the QTc interval.
Cabergoline^*^	D2 dopamine receptor agonist 0.5 mg, oral Dose: 0.5–7 mg weekly	Nausea Orthostatic hypotension Neurological and psychiatric symptoms Liver dysfunction	Azithromycin may be used with attention in patient with neurological and psychiatric symptoms and balance disorders. Azithromycin is ergot-derived; be careful in the association with other ergot derivatives like cabergoline. Lopinavir/Ritonavir plasmatic concentrations can increase in association with cabergoline.
**Drugs with perypheral adrenal action**			
Metyrapone^*^	Steroidogenesis inhibitor Blocks 11 beta hydroxylase 250 mg, oral Initial dose: 250–1,000 mg that must be titrated in the first month To avoid hypoadrenalism, this could be combined with replacement therapy	Hepatotoxicity Hypertension Hypokalemia Gastrointestinal discomfort Dizziness Hirsutism in women Adrenal crisis Edema Pneumocystis Jirovecii infection	Paracetamol hepatotoxic effect may be potentiated Hydroxychloquine and Chloroquine may be used with attention on hypokalemia
Ketoconazole	Steroidogenesis inhibitor Blocks the 11 beta, 17 alpha, 18 hydroxylase (inhibits synthesis of cortisol and also aldosterone and androgens) 200 mg, oral Initial dose: 200–600 mg/day Maximum dose: 800–1,200 mg/day Refracted doses: 2–3 times a day	Hepatotoxicity Gastrointestinal side effects Rash Gynecomastia Hypogonadism Adrenal insufficiency Liver dysfunction	Hydroxychloquine and Chloroquine may be used with attention on hypokalemia Hydroxychloquine, Chloroquine, Azithromycin, Lopinavir, and Ritonavir prolong QTc interval Metabolized by CYP3A4 cytochrome: Azithromycin and Lopinavir/Ritonavir may increase Ketoconazole concentration; Tocilizumab may decrease Ketoconazole concentration It is a CYP3A4 inhibitor and it can increase Glucocorticoid and Lopinavir/Ritonavir concentration Interaction with antiviral drugs like Ritonavir and antibiotic like Clarithromycine
Mitotane	Steroidogenesis inhibitor Adrenolytic action 500 mg, oral Dose: 0.5–3 g, three times a day	Hepatotoxicity GI discomfort Neurological disorders Hypothyroidism Hypogonadism	It is a CYP3A4 inhibitor and it can increase Glucocorticoid and Lopinavir/Ritonavir concentration Must be suspended in case of shock or infection
**Drug with action on glucocorticoid receptor**			
Mifepristone	Reversible blockade of glucocorticoid receptor In Italy, the prescription is limited to compassionate use 200 mg, oral Dose: 300–1,200 mg/day	Hypokalemia Worsening hypertension Adrenal crisis	Metabolized by CYP3A4 cytochrome: Azithromycin and Lopinavir/Ritonavir may increase Mifepristone concentration; Tocilizumab may decrease Mifepristone concentration Hydroxychloquine and Chloroquine may be used with attention on hypokalemia
**Drug with citotoxic action**			
Temozolomide^*^	Cytotoxic second generation alkylating agent by DNA methylation induces apoptosis by accumulation of alkylated substances 5, 20, 100, 140, 180, and 250 mg, oral Administered in cycles	Embryotoxic, Teratogenic, and Genotoxic Severe myelosuppression Pneumocystis Jirovecii infection Gastrointestinal side effects Asthenia Anorexia	Tocilizumab may induce myelosuppression and immunodepression

* *Denotes the medications that have been prescribed in the case report*.

We found that the ESE clinical guidance on Cushing's management during COVID-19 has been able to predict the possible clinical scenario that we evidenced in clinical practice. The guidance suggests for CS patients on long-term treatment who are clinically and biochemically stable with the same medication dose to be maintained in their current regimen (8). This condition does not completely apply to this case considering the recent reintroduction of metyrapone and the new chemotherapy schedule to control the pituitary mass and hypercortisolism. Switching to a “block and replace” regimen was suggested to allow a better control and to reduce the risk of adrenal insufficiency (8). In addition, education about the “sick day rules” and access to stress doses of glucocorticoid was underlined (8). Eventually, this patient developed the signs and symptoms of adrenal insufficiency, which determined the progressive suspension of CS therapies so that eventually a stress dose of i.v. hydrocortisone was needed. We suggested, at discharge, a careful monitoring of CS disease activity and a close endocrinological follow-up in order to ensure the early restart of previous treatments. Serum cortisol levels were not informative in this context as they have been probably overestimated owing to assay cross-reactivity with the precursor 11-deoxicortisol.

This case allowed us a reflection on other aspects related to COVID-19 infection in association with CS. CS patients might develop a severe form of COVID-19 infection, considering that the major comorbidities associated with COVID-19 mortality are hypertension, diabetes, previous heart disease, and cerebral infarction (10). Indeed, CS patients frequently present a combination of these comorbidities, especially diabetes and hypertension. All forms of diabetes expose patients to an increased risk of infection because of defects in innate immunity affecting phagocytosis, neutrophil chemotaxis, and cell-mediated immunity. Among the possible mechanisms, we emphasize that DPP-4 was identified as a functional receptor for human coronavirus and the treatment with DPP-4 inhibitors should not be suspended during the hospitalization because these agents could reduce DPP-4 concentrations, thus reducing the viral cell entry (16). In this case, sitagliptin was maintained as long as possible and was eventually suspended when glucose values were reduced in the context of hypoadrenalism.

It is well known that CS patients have a high risk to developing embolism, not only pulmonary but also cerebral. In particular, CS patients presenting a hypercoagulability state, which added to the demonstrated COVID-19 virus thrombotic risk, leads us to strongly consider a treatment with a prophylactic anticoagulant (17). Supported by previous reviews (18), we observed that this case, who had been on anticoagulant doses of enoxaparin for 2 months for the thrombosis of the upper right arm, had the tendency to develop hyperkalemia, which was associated to aldosterone levels toward the lower limit. Also, after the partial correction of hypoadrenalism, the potassium levels did not change significantly. Therefore, we hypothesized that additional factors were affecting potassium balance, such as heparin and heparinoids, which are reported to be among the reversible inhibitors of aldosterone production. In fact, heparin is a potent inhibitor of aldosterone production—this effect is specific for the zona glomerulosa and does not affect other corticosteroids—and reduces both plasma aldosterone concentration and urinary aldosterone excretion (18). Aldosterone suppression results in natriuresis and in decreased urinary excretion of potassium. Probably, the combination of long-term treatment with heparin associated to hypoadrenalism was responsible for the unexpected high potassium levels in this patient. Eventually, we managed to treat the thrombotic disorder with oral anticoagulants.

From the patient's perspective, both hypercorticism and adrenal insufficiency were experienced during the chronic and acute disease. In addition, the COVID-19 infection may be worsened by concomitant hypopituitarism that must be promptly recognized and treated.

## Teaching Points

→ Adrenal insufficiency might be a clinical challenge that needs a prompt treatment also in CS patients during COVID-19 infection.
→ Consider the possibility to titrate or temporary halt medical therapies for CS in the context of COVID-19 infection in order to avoid adrenal insufficiency.
→ Unexpected hyperkalemia in CS patients under heparin treatment might be a sign of aldosterone suppression.
→ Consider the many possible interactions between CS and COVID-19 medications.

## Data Availability Statement

The raw data supporting the conclusions of this article will be made available by the authors, without undue reservation upon reasonable request.

## Ethics Statement

Written informed consent was obtained from the individual(s) for the publication of any potentially identifiable images or data included in this article.

## Author Contributions

FBe, FBo, LB, RM, ED, and PM analyzed and interpreted the patient data. FBe, FD, MP, MB, CS, FF, RV, and PM were major contributors in writing the manuscript. All authors contributed to the article and approved the submitted version.

## Conflict of Interest

The authors declare that the research was conducted in the absence of any commercial or financial relationships that could be construed as a potential conflict of interest.
